# Severe refractory TAFRO syndrome requiring continuous renal replacement therapy complicated with *Trichosporon asahii* infection in the lungs and myocardial infarction: an autopsy case report and literature review

**DOI:** 10.1186/s41100-018-0157-8

**Published:** 2018-04-04

**Authors:** Arata Hibi, Ken Mizuguchi, Akiko Yoneyama, Takahisa Kasugai, Keisuke Kamiya, Keisuke Kamiya, Chiharu Ito, Satoru Kominato, Toshiyuki Miura, Katsushi Koyama

**Affiliations:** 1 Division of Nephrology and Rheumatology, Department of Internal Medicine, Kariya Toyota General Hospital, 5-15, Sumiyoshi-cho, Kariya, Aichi 448-8505 Japan; 2 Deaprtment of Pathology, Kariya Toyota General Hospital, 5-15, Sumiyoshi-cho, Kariya, Aichi 448-8505 Japan; 3 Division of Nephrology and Rheumatology, Department of Internal Medicine, Aichi Medical University Hospital, 1-1 Yazakokarimata, Nagakute, Aichi 480-1195 Japan

**Keywords:** TAFRO syndrome, Multicentric Castleman disease, Interleukin-6, Hypercytokinemia, Capillary leak syndrome, Renal failure, Endothelial injury, Thrombotic microangiopathy, Continuous renal replacement therapy, Sepsis-related myocardial calcification

## Abstract

**Background:**

TAFRO (thrombocytopenia, anasarca, fever, reticulin myelofibrosis/renal failure, and organomegaly) syndrome is a systemic inflammatory disorder and unique clinicopathological variant of idiopathic multicentric Castleman disease that was proposed in Japan. Prompt diagnosis is critical because TAFRO syndrome is a progressive and life threating disease. Some cases are refractory to immunosuppressive treatments. Renal impairment is frequently observed in patients with TAFRO syndrome, and some severe cases require hemodialysis. Histological evaluation is important to understand the pathophysiology of TAFRO syndrome. However, systemic histopathological evaluation through autopsy in TAFRO syndrome has been rarely reported previously.

**Case presentation:**

A 46-year-old Japanese man with chief complaints of fever and abdominal distension was diagnosed with TAFRO syndrome through imaging studies, laboratory findings, and pathological findings on cervical lymph node and bone marrow biopsies. Interleukin (IL)-6 and vascular endothelial growth factor (VEGF) levels were remarkably elevated in both blood and ascites. Methylprednisolone (mPSL) pulse therapy was initiated on day 10, followed by combination therapy with PSL and cyclosporine A. However, the amount of ascites did not respond to the treatment. The patient became anuric, and continuous renal replacement therapy was initiated from day 50. However, the patient suddenly experienced cardiac arrest associated with myocardial infarction (MI) on the same day. Although the emergent percutaneous coronary intervention was successfully performed, the patient died on day 52, despite intensive care. Autopsy was performed to ascertain the cause of MI and to identify the histopathological characteristics of TAFRO syndrome.

**Conclusions:**

Bacterial peritonitis, systemic cytomegalovirus infection, and *Trichosporon asahii* infection in the lungs were observed on autopsy. In addition, sepsis-related myocardial calcification was suspected. Management of infectious diseases is critical to reduce mortality in patients with TAFRO syndrome. Although the exact cause of MI could not be identified on autopsy, we considered embolization by fungal hyphae as a possible cause. Endothelial injury possibly caused by excessive secretion of IL-6 and VEGF contributed to renal impairment. Fibrotic changes in anterior mediastinal fat tissue could be a characteristic pathological finding in patients with TAFRO syndrome.

## Background

Castleman disease (CD), first described by Castleman et al. in 1956, is a rare lymphoproliferative disorder with angiofollicular lymph node hyperplasia [1]. CD is further separated into the following two distinct diseases: unicentric CD (UCD) and multicentric CD (MCD). Unlike UCD, MCD shows systemic inflammatory symptoms and multiple organ involvement owing to excessive secretion of proinflammatory cytokines, particularly interleukin-6 (IL-6) [2]. Human herpesvirus 8 (HHV-8) is a well-established cause of hypercytokinemia and is strongly associated with the pathogenesis of MCD [3]. Interestingly, HHV-8-encoded viral IL-6 was found to induce a MCD-like phenotype in an animal study [4]. HHV-8-negative MCD is currently referred to as idiopathic MCD (iMCD) [3]. According to the histopathological features of the lymph nodes in patients with iMCD, the disease was further divided into the following three groups: hyaline vascular (HV) type, plasma cell (PC) type, and mixed type [3]. In 2008, Kojima et al. assessed clinicopathological findings among Japanese patients with iMCD and classified the disease into the following two groups: idiopathic plasmacytic lymphadenopathy with polyclonal hyperimmunoglobulinemia (IPL) type and non-IPL type [5]. Non-IPL-type iMCD usually manifests as HV type or mixed type histologically and is frequently associated with ascites and pleural effusion in addition to autoimmune diseases during the clinical course [5]. TAFRO (thrombocytopenia, anasarca, fever, reticulin myelofibrosis/renal failure, and organomegaly) syndrome is a novel systemic inflammatory disease associated with the excessive release of IL-6, which was first described by Takai et al. in 2010 in the Japanese literature [6]. In 2011, Kojima et al. reported seven cases of Japanese patients diagnosed with iMCD, who showed effusions (Castleman-Kojima disease) [7]. TAFRO syndrome (Castleman-Kojima disease) was initially thought to be a variant of iMCD. In 2012, Japanese researchers discussed whether TAFRO syndrome is a distinct clinicopathological entity [8]. In 2015, Iwaki et al. performed clinicopathological analysis of TAFRO syndrome and proposed TAFRO syndrome as a distinct entity within the larger entity of iMCD. The authors proposed further subclassification of iMCD into TAFRO-iMCD and iMCD-NOS (not otherwise specified). In their study, anasarca, fever, and elevated alkali phosphatase (ALP) levels were significantly observed and hypergammaglobulinemia was infrequently observed in patients with TAFRO-iMCD when compared with patients having iMCD-NOS [9]. Proposed diagnostic criteria, disease severity classification, and treatment strategies for TAFRO syndrome were presented by Japanese research teams in 2015 [10]. TAFRO syndrome mainly occurs in Japanese patients in the fifth decade of life [10]; however, a case in a Japanese adolescent has been reported [11]. Prompt diagnosis and treatment are essential because TAFRO syndrome is usually aggressive and life threating.

Pathological evaluation is important for understanding the etiology of TAFRO syndrome. However, autopsy case reports of TAFRO syndrome have been rarely reported in the literature. Here, we present a case of a 46-year-old Japanese man with severe TAFRO syndrome requiring dialysis, which was refractory to the commonly used combination therapy with glucocorticoids and cyclosporine A (CsA) and was complicated with severe infections and ST-elevated myocardial infarction (STEMI) during the clinical course. We also present findings of a comprehensive review of the literature on TAFRO syndrome and report some characteristic histopathological findings on autopsy in the present case.

## Case presentation

**Fig. 1:**
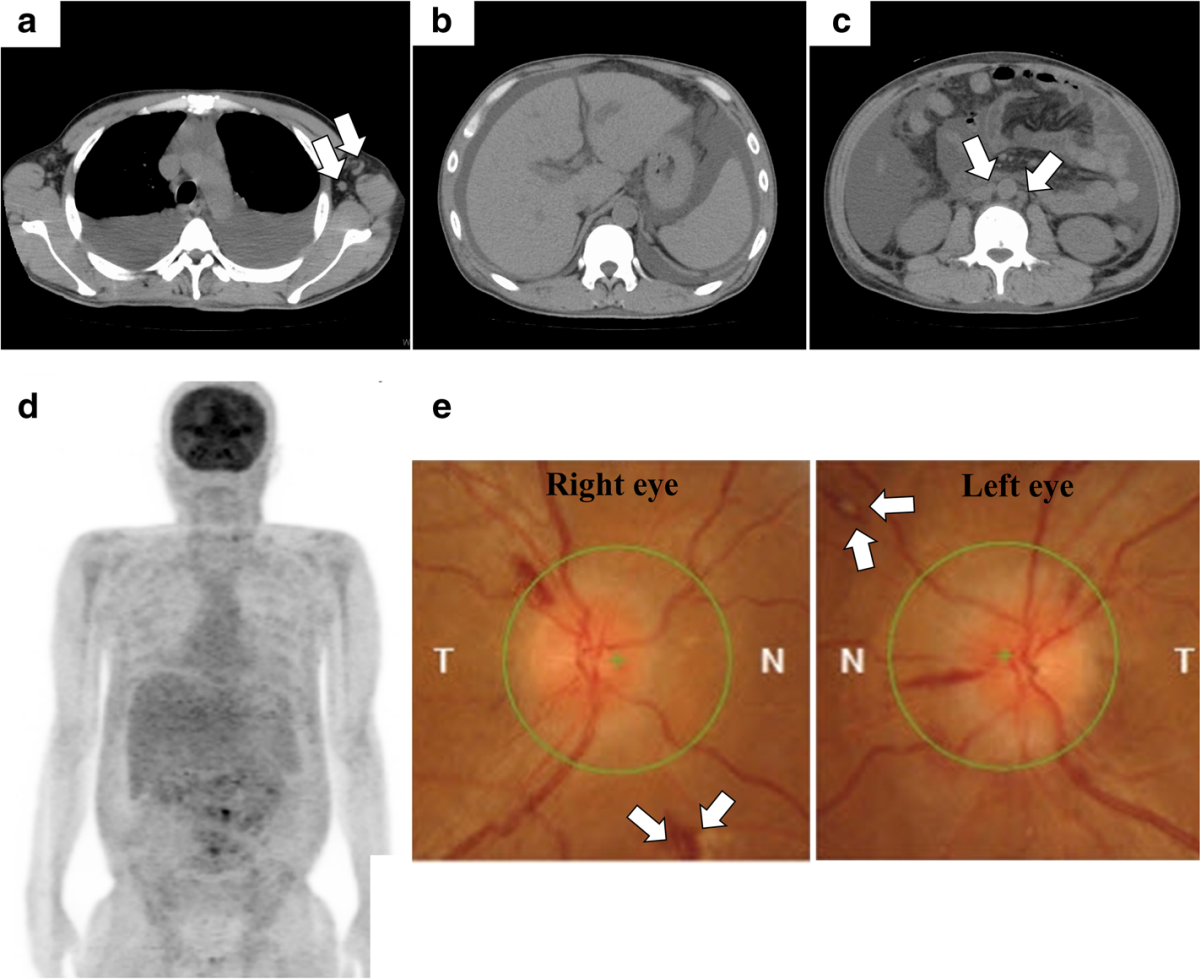
Imaging findings. **a**–**c** Computed tomography images on the day of admission. **a** Massive pleural effusion and slightly enlarged axillary lymph nodes are observed (arrows). **b** Hepatosplenomegaly is seen. **c** Massive ascites and slightly enlarged para-aortic lymph nodes are observed (arrows). **d** ^18^F–fluorodeoxy-glucose positron emission tomography (FDG-PET) images on day 8. Although the findings are poor (FDG uptake is generally weak), FDG uptake is observed in the para-aortic lymph nodes. **e** Funduscopic evaluation performed on day 10. Bilateral optic disk edema is remarkable. Roth’s spots are observed (arrows). Hemorrhage in the fundus of right eye is also observed

**Fig. 2:**
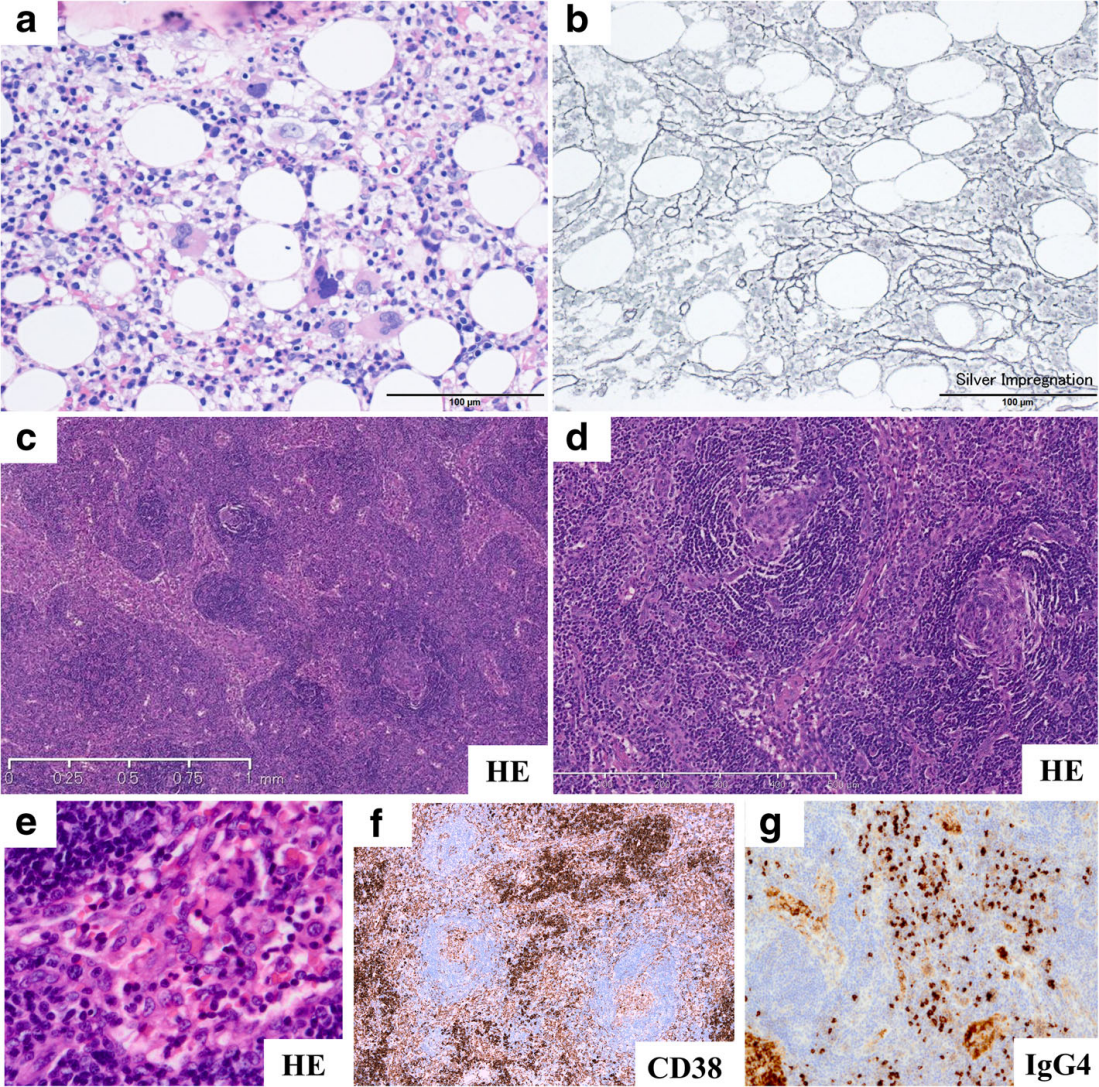
Pathological findings. **a**, **b** Pathological evaluation involving bone marrow biopsy in the present case performed on day 9. **a** Mild increased number of megakaryocytes is observed (hematoxylin and eosin [H&E] staining). **b** Mild reticulin myelofibrosis in bone marrow is observed (silver impregnation staining). **c**–**h** Pathological evaluation of the right neck lymph node in the present case. **c**, **d** Atrophic germinal center, vascular invasion with a glomerular-like pattern of vascular endothelial cell proliferation and hyalinization are observed in the follicles (H&E staining). **e** Dendric proliferation of arterioles and swelling of vascular endothelial cells are observed (H&E staining). **f**, **g** Invasion of plasma cells (CD38+) is observed in the intrafollicular space (immunohistochemical staining). **h** Immunoglobulin G (IgG) 4-positive plasma cells are observed (> 10 IgG4-positive plasma cells/high power field on immunohistochemical staining); however, the IgG4/IgG ratio is 24.2%, and it does not fulfill the criteria for IgG4-related disease (> 40%, data not shown). Human herpesvirus 8 is negative on immunohistochemical staining, and the Epstein-Barr virus-encoded small RNA in situ hybridization is negative in the lymph node (data not shown). These findings are compatible with mixed-type multicentric Castleman disease-like histology

**Fig. 3:**
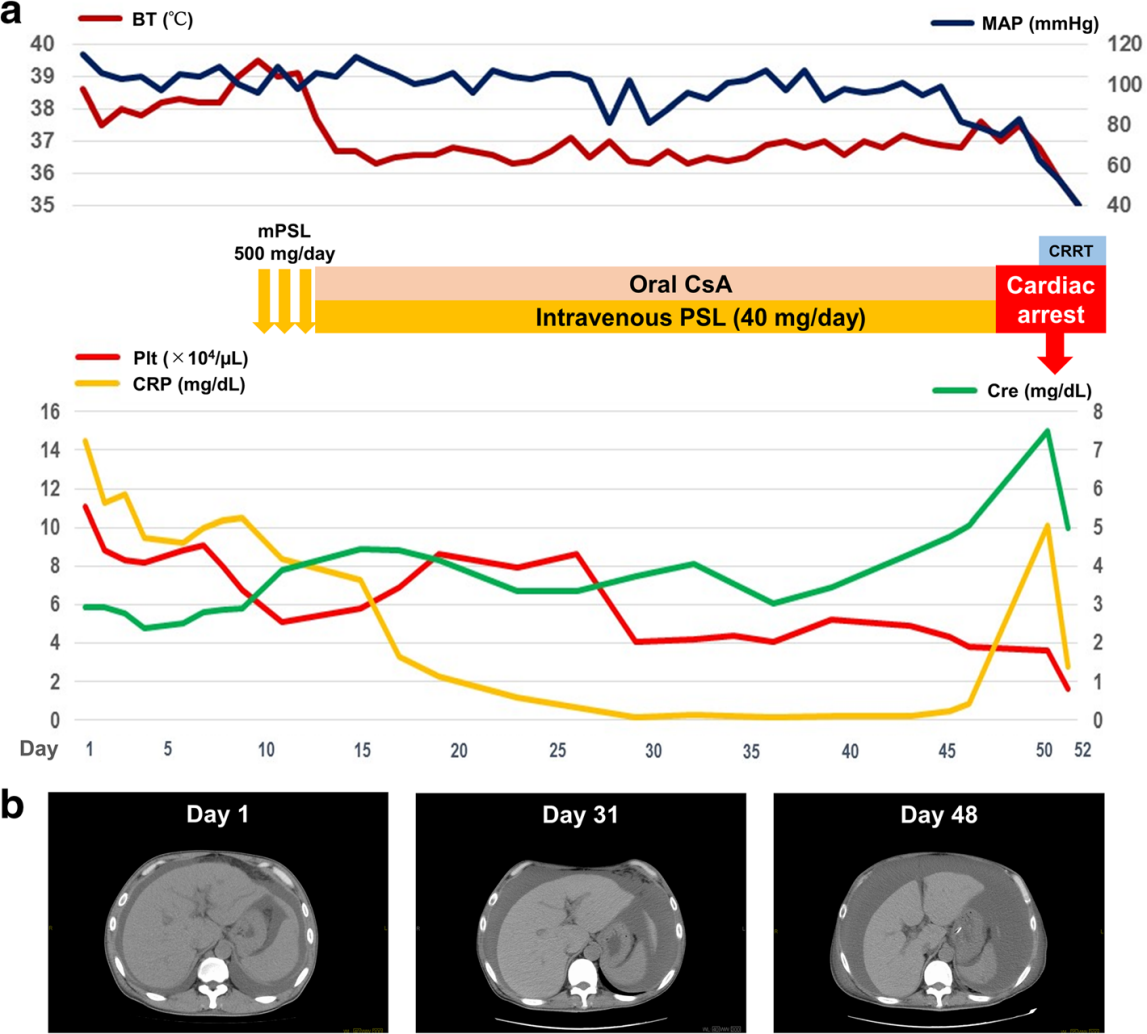
Clinical course of the present case. **a**, **b** The patient became afebrile after pulse methylprednisolone therapy. The C-reactive protein (CRP) level decreased to within the normal range with combination therapy involving intravenous glucocorticoid and oral cyclosporine A. However, despite the treatment, the amount of ascites increased gradually and renal impairment did not improve. The CRP and serum creatinine levels were elevated on day 50, and complicated infection was suspected. After the initiation of continuous renal replacement therapy, the patient experienced cardiac arrest on the same day because of myocardial infarction. Despite intensive care, including antibiotics therapy and continuous hemodiafiltration, the patient died on day 52. *BT* body temperature, *Cre* creatinine, *CRP* C-reactive protein, *CRRT* continuous renal replacement therapy, *CsA* cyclosporine A, *MAP* mean arterial pressure, *mPSL* methylprednisolone, *Plt* platelet

**Fig. 4:**
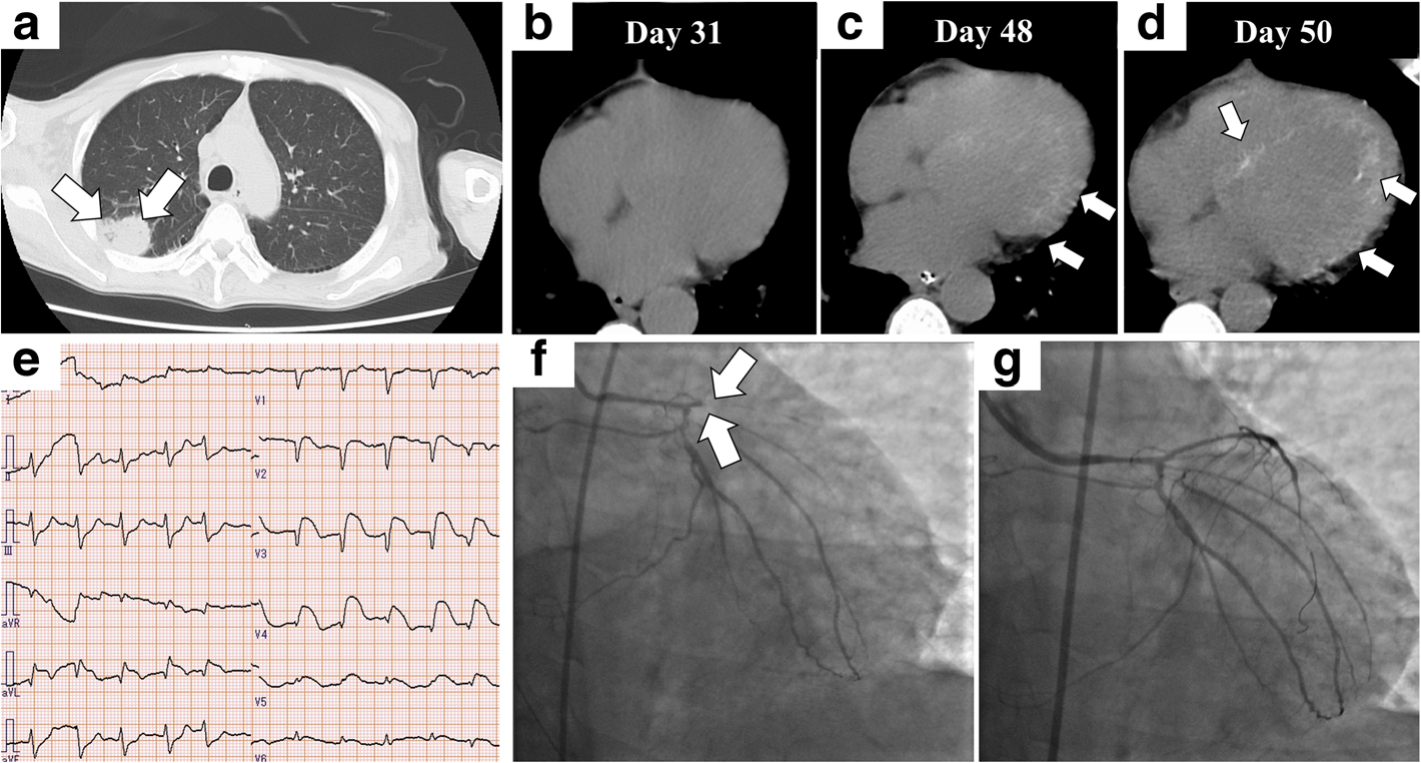
Imaging and assessments after treatment. **a** Computed tomography (CT) images of the chest on day 50. Pleural effusion resolved, but a mass lesion (arrows) is newly observed. **b**–**d** CT images of the heart on days 31, 48, and 50. Scattered appearance of high intensity is gradually seen in the cardiac wall. CT performed on day 31 shows no remarkable finding in the myocardium. However, high intensity gradually became apparent on CT images obtained on days 48 and 50 (arrows). **e**–**g** Electrocardiogram performed after recovery of spontaneous circulation and coronary angiography (CAG) results on day 50. **e** ST elevations are observed at leads V2–5 and aVL. Reciprocal changes are observed at leads I, II, and aVF on electrocardiography. **f** CAG shows complete occlusion of the left descending coronary artery (arrows). **g** After percutaneous old balloon angioplasty, reperfusion of blood flow is achieved

A 46-year-old Japanese man without a remarkable medical history visited our hospital with chief complaints of fever, fatigue, generalized edema, and abdominal distention. His fever started two weeks prior to admission. Abdominal distention and edema gradually worsened, and he gained 7 kg of weight within two weeks, despite low food intake owing to loss of appetite. He denied previous episodes of infectious diseases. At initial presentation, he appeared exhausted. His vital signs were as follows: body temperature, 37.8 °C; blood pressure, 160/93 mmHg; heart rate, 109 beats/min; respiratory rate, 22 breathes/min; and oxygen saturation, 90% with room air. On physical examination, generalized pitting edema was observed. His heart sounds were normal, but his lung sounds were weak at the lung base on both sides. Jaundice was not observed, but a distended abdomen and hepatomegaly were observed. Blood test results on the day of admission revealed an elevated white blood cell (WBC) count (14,600/μL), mild anemia (hemoglobin level, 11.1 g/dL), thrombocytopenia (platelet count, 11.1 × 10^4^/μL), renal impairment (blood urea nitrogen level, 60.7 mg/dL and serum creatinine level, 2.94 mg/dL), an elevated C-reactive protein (CRP) level (14.25 mg/dL), an elevated ALP level (768 U/L), polyclonal hypergammaglobulinemia (immunoglobulin G [IgG], 2461 mg/dL), and an elevated IgG4 level (235 mg/dL). Immunological screening test results for autoantibodies were negative, except for positive antinuclear antibody (× 40) and anti-SS-A antibody. Polymerase chain reaction for HHV-8 DNA in a serum sample was negative (Table 1). Computed tomography (CT) performed on the day of admission revealed massive pleural effusions and ascites, generalized mild lymphadenopathy (< 1.5 cm in diameter), and hepatosplenomegaly (Fig. 1a–c). Echocardiography performed on day 2 revealed normal wall motions without any sign of valvular disease, but a collapsed inferior vena cava was observed (maximum diameter < 5 mm). ^18^F–fluorodeoxyglucose positron emission tomography (FDG-PET) revealed no apparent FDG uptake, except for slight uptake in the para-aortic lymph nodes (Fig. 1d). Considering the positive anti-SS-A antibody finding, lip biopsy was performed on day 6, but the pathological findings did not meet the criteria for Sjögren syndrome (SjS). On ophthalmological examination, keratoconjunctivitis was not observed; however, bilateral optic edema was remarkable (Fig. 1e). Serum IL-6 and plasma VEGF levels were assessed in a blood sample obtained on day 9, and both were elevated (25.2 pg/mL and 224 pg/mL, respectively). Abdominal paracentesis was performed on the same day, and the levels of IL-6 and vascular endothelial growth factor (VEGF) in ascites were remarkably high (3310 pg/mL and 335 pg/mL, respectively). Bone marrow examination was performed on day 9. Bone marrow aspiration was dry tap, and bone marrow biopsy revealed a mild increase in megakaryocytes and mild reticulin myelofibrosis on silver impregnation staining (Fig. 2a, b). Biopsy of a cervical lymph node was performed on day 10, and the pathological findings were compatible with a mixed-type MCD histology (Fig. 2c–g). We suspected TAFRO syndrome and methylprednisolone (mPSL) pulse therapy (500 mg/day for three consecutive days) was initiated on day 10 after lymph node biopsy, followed by intravenous PSL (40 mg/day) and oral CsA administration. The disease severity of TAFRO syndrome one day prior to treatment initiation was very severe (grade 5) [10]. After the initiation of immunosuppressive treatment, the patient became afebrile and the CRP level returned to the normal range within two weeks. However, the patient’s platelet count and serum creatinine level as well as his overall condition did not improve, even after the initiation of immunosuppressive treatment (Fig. 3a). A central venous catheter was inserted and total parenteral nutrition was started as he could not eat owing to loss of appetite and refusal of tubal feeding. Diuretics, including furosemide, potassium canrenoate, trichlormethiazide, and tolvaptan were administered during hospitalization; however, the amount of ascites did not decrease (Fig. 3b). Urinalysis performed on day 25 revealed mild proteinuria (0.34 g/day) with a fractional excretion of sodium of 5.1% and a fractional excretion of urea nitrogen (FE_UN_) of 20.7%. Because urinalysis was performed under diuretics use, considering FE_UN_ level and intravascular hypovolemia on echography, intravenous fluid replacement was performed to correct the pre-renal factor responsible for renal impairment. However, the patient’s volume depletion and blood pressure did not respond well to intravenous fluid replacement. Abdominal paracentesis was again performed on day 40, and it revealed persistently high levels of IL-6 and VEGF (4320 pg/mL and 421 pg/mL, respectively). Although urine volume was initially preserved with diuretic administration, the patient became oliguric from day 47 and anuric from day 49. He was hemodynamically unstable on day 50 (body temperature, 37.5 °C; blood pressure, 84/52 mmHg; heart rate, 109 beats/min; respiratory rate, 24 breaths/min; oxygen saturation, 95% with room air). As the patient was undergoing immunosuppressive therapy and his CRP levels increased by 10.08 mg/dL on the same day, systemic examination was performed to rule out the possibility of infectious disease. Analysis of ascites was performed on the same day, revealing purulent ascites, and *Escherichia coli* was isolated. *E. coli* was also isolated from blood cultures. CT of the chest showed a mass in the right lung (Fig. 4a) in addition to scattered appearance of high intensity regions in the myocardium, which were newly observed (Fig. 4b–d). Blood tests performed on day 50 revealed elevated β-d-glucan levels (23.0 pg/mL) and positive cytomegalovirus (CMV) antigenemia (41 cells/5 × 10^4^ WBCs on the C7-HRP test). *Aspergillus*, *Candida mannan*, and *Cryptococcus neoformans* antigens were negative. We suspected bacterial peritonitis and fungal pneumonia. Intravenous meropenem, vancomycin, and caspofungin were initiated on the same day. Continuous renal replacement therapy (CRRT) using a polymethyl-methacrylate membrane was also initiated on the same day because anuria did not improve and the patient was hemodynamically unstable. Conditions of CRRT were as follows: mode, continuous hemodiafiltration; dialysis membrane, CH-1.8 W (Toray Medical Co., Ltd., Tokyo Japan); and anticoagulant, nafamostat mesilate. The blood, dialysate, substitute, and filtration flow rates were 100 mL/min, 400 mL/h, 400 mL/h, and 800 mL/h, respectively.

**Table 1:** Laboratory findings of the present case

Complete blood count		Blood urea nitrogen	60.7 mg/dL	Serum M protein	Negative
White blood cell	14,600 /μL	Creatinine	2.94 mg/dL	Platelet-associated IgG	238.0 ng/10^7^ cells
Neutrophil	84.6%	C-reactive protein	14.25 mg/dL	Anti-platelet Ab	Negative
Lymphocyte	9.4%	β-D-glucan	< 6.0 pg/mL	Soluble IL-2 receptor	1120 U/mL
Monocyte	5.6%	Ferritin	419 ng/mL	Direct Coombs test	Negative
Basophil	0.1%	Serum iron	9 μg/dL	Indirect Coombs test	Negative
Eosinophil	0.0%	Total iron binding capacity	155 μg/dL	IL-6 (serum, on day 9)	25.2 pg/mL
Red blood cell	426 × 10^4^ /μL	Haptoglobin	268 mg/dL	VEGF (plasma, on day 9)	224 pg/mL
Hemoglobin	11.7 g/dL	Thyroid stimulating hormone	6.4 μIU/ml	IL-6 (ascites, on day 9)	3310 pg/mL
Hematocrit	34.8%	Free T3	1.4 pg/ml	VEGF (ascites, on day 9)	335 pg/mL
Reticulocyte	15‰	Free T4	1.1 ng/dl	STS	Negative
Platelet	11.1 × 10^4^ /μL	BNP	61 pg/mL	HBs Ag	Negative
IPF	10.9%	CEA	0.5 U/mL	HBs Ab	Negatve
Coagulation test		CA19–9	5 ng/mL	HBc Ab	Negative
APTT	45.3 s	IgA	264 mg/dL	HCV Ab	Negative
PT-INR	1.30	IgG	2461 mg/dL	HIV Ab	Negative
Fibrinogen	479 mg/dL	IgG4	235 mg/dL	IFN-γ release assay	Negative
Blood chemistry and immunological tests		IgM	88 mg/dL	* Helicobacter pylori* IgG	Negative
Sodium	138 mEq/L	ANA	Positive (×40)	CMV-IgG	Positive
Potassium	4.1 mEq/L	RF	2 U/mL	CMV-IgM	Negative
Chloride	101 mEq/L	CH50	44.9 U/mL	EBV VCA-IgG Ab	Positive
Calcium	8.1 mg/dL	C3	50 mg/dL	EBV VCA-IgM Ab	Negative
Total protein	6.4 g/dL	C4	14 mg/dL	EBNA Ab	Positive
Albumin	2.1 g/dL	Anti-ds-DNA IgG Ab	Negative	HHV-8 DNA PCR	Negative
Total bilirubin	0.7 mg/dL	PR3-ANCA	Negative	Bacterial cultures	
Asparate aminotransferase	28 U/L	MPO-ANCA	Negative	Blood cultures	Negative
Alanine aminotransferase	12 U/L	Anti-SS-A Ab	> 1200 U/mL	Ascitic fluid cultures	Negative
Lactate dehydrogenase	278 U/L	Anti-SS-B Ab	Negative	Urine culture	Negative
Alkali phosphatase	768 U/L	Anti-CL Ab	Negative	Urinalysis	
γ- glutamyltransferase	129 U/L	Anti-CLβ_2_GPI Ab	Negative	Protein	(1+)
Amylase	50 U/L	Anti-Scl-70 Ab	Negative	Occult blood	(−)
Creatine kinase	694 U/L	Anti-RNP Ab	Negative	NAG	44.8 U/L
Glucose	121 mg/dL	Anti-Sm Ab	Negative	β2-MG	81 μg/L
Hemoglobin A1c	5.3%	Anti-mitochondrial M2 Ab	Negative	Granular casts	10–19 /WF
Uric acid	15.1 mg/dL	Anti-smooth muscle Ab	Negative	BJP	Negative

*β2-MG* β2-microglobulin. *Ab* antibody, *ANA* antinuclear antibody, *APTT* activated partial thromboplastin time, *BJP* Bence Jones protein *BNP* brain natriuretic peptide, *C3* complement component 3, *C4* complement component 4, *CA 19–9* carbohydrate antigen 19–9, *CEA* carcinoembryonic antigen, *CH50* 50% hemolytic complement activity, *CL* cardiolipin, *CMV* cytomegalovirus, *ds-DNA* double stranded-DNA, *EBNA* Epstein-Barr virus-nuclear antigen, *EBV* Epstein-Barr virus, *GPI* glycoprotein I, *HBc Ab* hepatitis B core antibody, *HBs Ag* hepatitis B surface antigen, *HCV* hepatitis C virus, *HHV-8* human herpes virus-8, *HIV* human immunodeficiency virus, *IFN-γ* interferon-γ, *Ig* immunoglobulin, *IL* interleukin, *IPF* immature platelet fraction, *MPO* myeloperoxidase, *NAG* N-acetyl-β-D-glucosaminidase, *PCR* polymerase chain reaction, *PR3-ANCA* proteinase-3-anti-neutrophil cytoplasmic antibody, *PT-INR* prothrombin time-international normalized ratio, *RF* rheumatoid factor, *RNP* ribonucleoprotein, *Scl* scleroderma, *Sm* Smith, *SS* Sjögren syndrome, *STS* serologic test for syphilis, *T3* triiodothyronine, *T4* thyroxin, *TSH* Thyroid stimulating hormone, *VEGF* vascular endothelial cell growth factor, *VCA* viral capsid antigen, *WF* whole field

However, approximately 10 h after CRRT initiation, cardiac arrest (ventricular fibrillation followed by pulseless electrical activity) suddenly occurred. Return of spontaneous circulation (ROSC) was achieved by cardiopulmonary resuscitation with epinephrine injections. STEMI was suspected from electrocardiography findings obtained immediately after ROSC (Fig. 4e). Echocardiography performed immediately after ROSC revealed a generalized hypokinesis and akinesia of the anterior wall, septum, and apex without any apparent calcification of the cardiac wall. Blood tests performed after ROSC revealed a slightly elevated serum creatine kinase-MB level (80 U/L, normal range: < 25 U/L) and a remarkably elevated troponin I level (1.2904 ng/mL, normal range: < 0.0262 ng/mL), indicating the acute phase of MI. Emergent coronary angiography (CAG) was performed on the same day. On CAG, complete occlusion of the left anterior descending coronary artery was observed, and reperfusion was achieved through percutaneous old balloon angioplasty (Fig. 4f, g). An intra-aortic balloon pump was inserted to increase coronary blood flow and improve cardiogenic shock. However, even after successful coronary intervention, the patient was still hemodynamically unstable. Despite intensive care, the patient died on day 52.

**Fig. 5:**
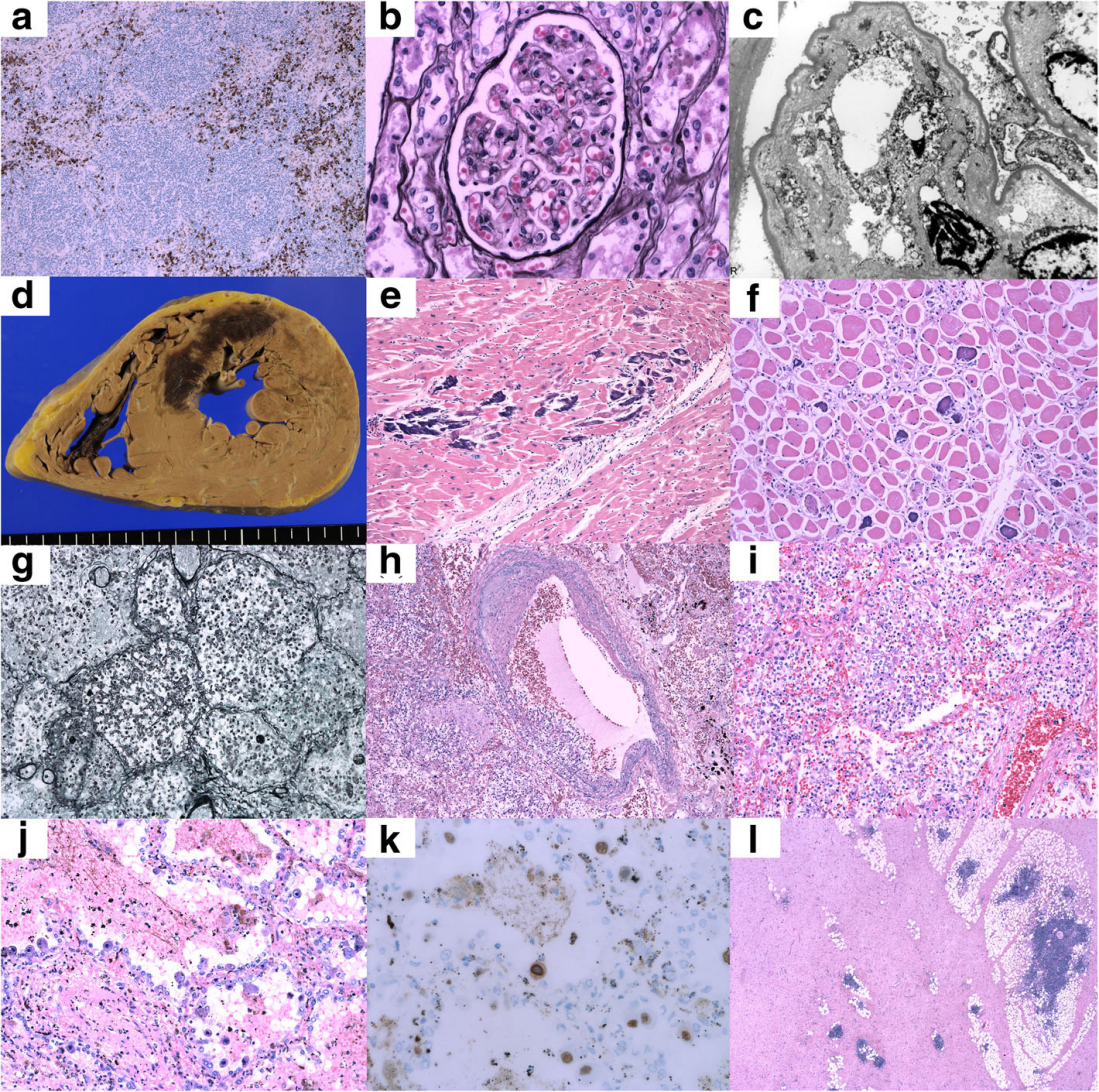
Histological findings on autopsy. **a** In lymph nodes, infiltration of CD38+ plasma cells was observed in the intrafollicular space (immunohistochemical staining of CD38). However, the number of plasma cells is decreased compared with that before treatment (Fig. 1f). **b** Double contour of the glomerular basement membrane with mild mesangiolysis is observed in the glomeruli (periodic acid-methenamine silver staining). **c** Subendothelial swelling is observed (electron microscopy). **d** Macroscopic appearance of a cross section of the heart revealed necrotic areas with dark reddish-brown color, corresponding to the anterior wall and septum. Left ventricle walls were thickened, and cardiac hypertrophy was observed (weight, 460 g). **e** Calcification is observed most remarkably in myocardial cells. Some of the myocardial cells are replaced by calcified tissue, and the remaining myocardial cells are intact (hematoxylin and eosin [H&E] staining). **f** Calcification is also observed in the skeletal muscle cells of the diaphragm (H&E staining). **g** In the right lung, fungal hyphae are abundant (Grocott staining). **h** In the lungs, intravascular invasion by fungal hyphae is observed (H&E staining). **i** Intra-alveolar invasion by fungal hyphae is observed (H&E staining). **j** Enlarged cells with intra-nuclear inclusion are remarkably observed in the lungs (H&E staining). **k** These cells are positive for cytomegalovirus (CMV) infection (immunohistochemical staining of CMV). **l** Subcutaneous tissue of the anterior mediastinum shows that fat tissue is largely replaced by fibrotic tissue. No infiltration of inflammatory cells is observed, and no remarkable finding in tissue of the thymus is noted (H&E staining)

Autopsy was performed after obtaining consent from the patient’s relatives. The histological findings of autopsy are shown in Fig. 5. In this case, multiple infections, including bacterial peritonitis (small abscesses were observed in the abdominal space, and gram-positive cocci and gram-negative rods were identified), systemic CMV infection (including the lungs, pancreas, and adrenal glands), and *Trichosporon asahii* infection in the lungs (*T. asahii* was isolated from sputum culture) were observed. Intravascular invasion of fungal hyphae was noted in the lungs. On histological evaluation of the heart, coagulation necrosis and neutrophil infiltration were observed, findings that were compatible with MI that occurred a few days before the autopsy. Considering that intravascular invasion of fungal hyphae was observed, embolization caused by fungal hyphae was a possible cause of MI in this case. Necrotic changes were observed in most internal organs. We considered multiple organ failure due to multiple severe infections and circulatory failure due to MI as the main causes of death in this patient. Although there was no stenotic lesion and vascular calcification was not apparent in the left anterior descending coronary artery, myocardial calcification was noted. Calcification in the skeletal muscle cells of the diaphragm and iliopsoas muscle without vascular calcification was also noted. The pathological findings of the kidney were compatible with thrombotic microangiopathy (TMA) and endothelial injury was noted, although fibrin thrombi were not observed in arterioles. IgA, IgG, and IgM were negative on immunofluorescence. Tubulointerstitial changes could not be evaluated because most of the tissue was necrotic due to changes after death and circulatory failure prior to death. In the bone marrow reevaluation, the number of megakaryocytes did not increase, and reticulin myelofibrosis was not remarkably changed. In the lymph node reevaluation, infiltration of plasma cells was decreased, and immunosuppressive therapy appeared partially effective, although the amount of ascites and IL-6 levels in ascites did not decrease in the present case. Fibrotic changes in the fat tissue of the anterior mediastinum were noted.

## Discussion

TAFRO syndrome was initially reported mainly from Japan, but recently, cases of TAFRO syndrome are being reported worldwide [12, 13]. As severe cases of iMCD-NOS might show TAFRO syndrome-like clinical manifestations, some cases of TAFRO syndrome might be diagnosed as severe iMCD-NOS [11]. Coutier et al. reviewed differences in clinical manifestations and treatment strategies in TAFRO syndrome between Japanese patients (32 cases) and non-Japanese patients (12 cases). They found that Japanese patients were significantly older than non-Japanese patients (mean age, 52.0 vs. 36.9 years); serum creatinine levels on admission were significantly higher in non-Japanese patients, and glucocorticoid therapy was significantly more frequent in Japanese patients. However, no significant differences in histopathological findings were observed between Japanese and non-Japanese patients [13]. Although treatment strategies for TAFRO syndrome are still based on previous clinical experiences, glucocorticoids are commonly used and positioned as the first-line therapy for TAFRO syndrome in Japan [10]. CsA is also a treatment choice if glucocorticoids alone are not effective [10]. Combination therapy with glucocorticoids and CsA was effective in previously reported cases [14, 15]. Considering the mechanism of action of CsA, IL-2 may play a pivotal role in TAFRO syndrome in addition to IL-6 [14]. However, we should be aware of the risk of calcineurin inhibitor nephrotoxicity when using CsA in patients with TAFRO syndrome. The induction of the vasoconstriction of the afferent arterioles by CsA could contribute to the reduction of glomerular filtration rate (GFR) [16] in addition to intravascular hypovolemia due to capillary leak syndrome in patients with TAFRO syndrome. Tocilizumab (TCZ) could be used safely in patients with renal impairment, and it might be a reasonable choice considering that excessive IL-6 secretion is one of the main pathophysiological events in TAFRO syndrome. Fujiki et al. reported a case of TAFRO syndrome complicated with severe infection during corticosteroid therapy, which was successfully treated with TCZ alone [17]. However, cases of TCZ-resistant TAFRO syndrome have been reported [15, 18]. Clinical experiences of using rituximab, cyclophosphamide, and thalidomide have been reported from Japan [19–21] In the present case, as combination treatment with PSL and CsA appeared ineffective even after 5 weeks, and TCZ was considered an alternative immunosuppressant. However, the patient’s condition became unstable before TCZ administration was initiated.

To understand the pathophysiology, analyzing cytokine profiles and their products in patients with TAFRO syndrome is important. Recently, Iwaki et al. have performed serum analyses and have noted significantly higher levels of interferon γ-induced protein 10 kDa (IP-10) in patients with TAFRO syndrome than that in those with PC-type iMCD; they have also suggested the involvement of IP-10 in the pathogenesis of TAFRO syndrome [22].

Because evidence-based treatment has not been established and indications for evaluating clinical response have not been well defined, the treatment of TAFRO syndrome is difficult. In the present case, the serum CRP level decreased within the normal range after two weeks of starting immunosuppressive therapy. However, the amount of ascites did not respond to the treatment, and the IL-6 and VEGF levels in ascites increased. Although the serum CRP level could be used for indicating initial severity, the CRP level alone might not be a good indicator for treatment response. The IL-6 level in ascites might reflect the refractoriness to treatment, considering the clinical course of the present case. VEGF levels in patients with TAFRO syndrome could be useful for evaluating treatment response, considering that the plasma VEGF level can be used for monitoring disease activity among patients with POEMS (polyneuropathy, organomegaly, endocrinopathy, monoclonal gammopathy, and skin changes) syndrome [23].

Imaging studies have important roles for understanding the pathophysiology of TAFRO syndrome. Sakashita et al. first reported the resolution of an anterior mediastinal mass in a patient with TAFRO syndrome after treatment with TCZ in addition to corticosteroid, and they proposed an association between the anterior mediastinal mass and TAFRO syndrome [24]. Ozawa et al. reported characteristic anterior mediastinal masses, which predominantly showed fat attenuation on CT images, in two cases of TAFRO syndrome [25]. In their report, the pathological evaluation of anterior mediastinal lesions revealed irregular fibrosis and lymphoplasmacytic infiltration [25]. Although an anterior mediastinal mass was not apparent in CT images in the present case, fibrosis in anterior mediastinal fat tissue was apparent, similar to their report. Nagano and Matsumoto reported the first case of TAFRO syndrome in which complete resection of a large anterior mediastinal mass was performed, and pathological findings indicated fibrous tissue with scattered lymph nodes [26]. Kiguchi et al. analyzed CT images in 11 patients with TAFRO syndrome and reported that matted appearance of an enlarged mediastinum was a characteristic CT finding of TAFRO syndrome [27]. An anterior mediastinal lesion could be associated with the etiology of TAFRO syndrome, and fibrous changes might correspond with inflammatory reactions associated with hypercytokinemia. Nakamura et al. reported diffuse hypointensity of the bone marrow in T1- and T2-weighted magnetic resonance images in patients with TAFRO syndrome, and they assumed that these findings reflected a reduction in the fatty component corresponding to reconversion into hematopoietic marrow owing to anemia and thrombocytopenia [28]. Behnia et al. mentioned the efficacy of FDG-PET for selecting a lymph node for biopsy, for ruling out malignancies, and for evaluating treatment response [29]. However, in the present case, biopsy of the right cervical lymph node, which did not show high uptake of FDG, was sufficient for pathological evaluation. As superficial lymph node biopsy is usually affordable, even in cases of severe TAFRO syndrome, it could be a useful examination for diagnosing TAFRO syndrome, even if the lymph node does not show FDG uptake on FDG-PET imaging. Considering that reevaluation of systemic lymph nodes in the present autopsy showed the partial resolution of the appearance of MCD, lymph node biopsy prior to treatment is recommended. Although ophthalmological findings of TAFRO syndrome have been rarely reported, Oritz et al. first reported that optic disk edema could be an ophthalmological finding in TAFRO syndrome [30]. In addition to optic disk edema, Roth’s spot was observed in the present case on fundus examination. Roth’s spot can be observed in a variety of diseases but is rarely observed in patients with MCD [31], and it could be one of the ophthalmological manifestations of TAFRO syndrome.

According to the diagnostic criteria for TAFRO syndrome, exclusion of autoimmune diseases is required [10]. It should be noted that systemic lupus erythematosus (SLE) sometimes manifests the same characteristics as TAFRO syndrome. Hasegawa et al. examined patients with SLE who met the criteria for TAFRO syndrome and found that older patients with SLE (onset ≥ 50 years of age) had clinical features similar to those of TAFRO syndrome [32]. Although anti-SS-A antibody was positive and IgG4 was elevated in the present case, the patient did not meet the criteria for either SjS or IgG4-related disease. We found cases of a 72-year-old Japanese man with positive anti-SS-A antibody and a 50-year-old Japanese woman with positive anti-SS-A and anti-SS-B antibodies without indicative symptoms of SjS [20, 33], a case of a 25-year-old Japanese woman with possible TAFRO syndrome having primary SjS [34], and a case of a 46-year-old Japanese woman who was diagnosed with SjS during hospitalization [35]. A previous report of a case of an 81-year-old French man with TAFRO syndrome that manifested positive anti-SS-A and anti-SS-B antibodies, who was diagnosed with SjS during hospitalization, has been published [36]. However, the association between SjS syndrome or anti-SS-A/SS-B antibodies with TAFRO syndrome remains unclear. Non-Japanese cases of TAFRO syndrome with positive autoantibodies, including anti-thyroid peroxidase antibody and anti-cardiolipin antibody, have been reported [12]. We could not identify a case of TAFRO syndrome involving significantly elevated IgG4 levels (≥135 mg/dL), displaying an association with IgG4-related disease (IgG4-RD). Although IgG4-RD was denied according to the histopathological evaluation in the present case, differentiating IgG4-RD from CD is sometimes challenging [37]. Sato et al. have analyzed six cases of MCD with abundant IgG4-positive cells, meeting the criteria of IgG4-RD (IgG4/IgG-positive cell ratio, > 40%), and they have concluded that serum IL-6 and CRP elevation was more frequent in patients with MCD than in those with IgG4-RD [38]. Clinically, a favorable treatment response to PSL therapy may indicate a greater possibility of IgG4-RD than that of MCD. [39] Only few studies have reported IgG4-RD with elevated serum IL-6 levels [39–41], and the role of IL-6 in patients with IgG4-RD remains unclear. As IgG4 levels have not been reported for all previous cases of TAFRO syndrome, further studies are needed to discuss the association between TAFRO syndrome and elevated IgG4 levels.

Infectious diseases should be excluded in the diagnosis of TAFRO syndrome; however, Simons et al. reported the first case of TAFRO syndrome complicated with Epstein-Barr virus infection [42]. Malignancies also should be ruled out in the diagnosis of TAFRO syndrome, but the risk of developing malignant lymphoma during the clinical course of TAFRO syndrome should be considered. Ohya et al. reported a case of diffuse large B-cell lymphoma during treatment for TAFRO syndrome [43], and hypercytokinemia might be associated with the development of malignant lymphoma.

Although a grading score was proposed in the diagnostic criteria for TAFRO syndrome [10], the prognostic factors of TAFRO syndrome have not been analyzed. Renal involvement is one of the most serious complications of TAFRO syndrome. Some severe cases might require temporary hemodialysis (HD). We reviewed previously reported cases of TAFRO syndrome requiring HD during the clinical course in the English literature and identified 19 cases (16 Japanese cases [17, 19, 20, 28, 33, 43–51] and 3 non-Japanese cases [42, 52, 53]). Japanese patients were older than non-Japanese patients (mean age, 57.6 vs. 35.7 years). Two patients (10.5%) died during the clinical course [28, 47]. Steroid was used in 18 cases (94.7%), and mPSL pulse therapy was used in 17 cases (89.5%). Only 5 cases (26.3%) were successfully treated with steroid therapy alone [28, 43, 49, 52]. It should be noted that almost all cases (84.2%) required HD within three weeks of admission. However, Tsurumi et al. have reported a case of recurrent TAFRO syndrome that required HD more than 20 months after the initial admission [33]. We should be aware of the recurrence and the exacerbation of TAFRO syndrome even after remission is achieved. Respiratory failure requiring mechanical ventilation, multiple organ failure, and severe infections caused by bacteria and CMV were major complications (Table 2). We should be aware that renal involvement in patients with TAFRO syndrome is rapidly progressive even after treatment initiation. Renal impairment might be an important prognostic factor in patients with TAFRO syndrome because a previous large study of iMCD revealed that renal dysfunction (estimated GFR ≤ 60 mL/min/1.73 m^2^) was an independent risk factor of mortality [54]. Investigating renal pathology is important for understanding the pathophysiology of TAFRO syndrome. However, only a few case reports with kidney biopsy results have been published in the literature [49, 53, 55, 56]. Performing kidney biopsy during the acute phase of TAFRO syndrome could be difficult because of the presence of massive ascites, presence of thrombocytopenia, and difficulty with placement in the prone position because of dyspnea. Tanaka et al. focused on renal involvement in TAFRO syndrome and reported that lobular accentuation, mesangiolysis, and double contour of the glomerular basal membrane associated with endothelial swelling (membranoproliferative glomerulopathy-like lesions), which are compatible with endothelial injury, were usually observed in thrombotic microangiopathy (TMA) [49]. In the present case, although thrombi were absent in glomerular capillaries and arterioles, pathological evaluation was compatible with TMA. However, our case had a limitation because complicated sepsis could also result in the manifestation of TMA in renal pathology. In previous renal biopsy analyses of patients with iMCD, TMA was observed in about 57.0% of the patients [57, 58]. TMA is also one of the manifestations of renal pathology in patients with POEMS syndrome [59]. The increased levels of serum IL-6 and VEGF could increase permeability of the arteriolar wall and glomerular capillaries and could be associated with endothelial injuries in patients with TAFRO syndrome. Although mild proteinuria can be observed in patients with TAFRO syndrome, Nakamori et al. have recently reported the first case of TAFRO syndrome manifesting nephrotic syndrome [56]. In their case, manifestations of TMA and double contour were absent although endothelial swelling, mild interstitial inflammation, fibrosis, and tubular atrophy were observed. The accumulation of more cases will be needed to discuss the pathophysiology of renal impairment in TAFRO syndrome and its pathological differences from iMCD-NOS. In patients with TAFRO syndrome, we should also aware of the fact that decreased intravascular volume due to capillary leak syndrome associated with hypercytokinemia and increased intra-abdominal pressure due to massive ascites could be predisposing factors for renal impairment [60, 61]. Okumura et al. reported a patient with TAFRO syndrome who showed cardiac arrest because of abdominal compartment syndrome caused by massive ascites and hypovolemia [50]. Although the amount of ascites increased after drainage in this patient with TAFRO syndrome, drainage of ascites could be a good indication in selected cases.

**Table 2:** Previously reported cases of TAFRO syndrome required hemodialysis during clinical course and the present case

Case number	[Reference] Reported year	Age /Sex	Underlying disease	Immunity anomalies	IL-6 (pg/mL)	LN histology	Durations from admission to initiation of HD	Immunosuppressive treatment	Complications	Renal outcome	Clinical outcome
Case 1	[44] 2013	56/M	ITP *H. pylori* infection	Platelet-associated anti-GPIIb/IIIa Ab	7.2 (serum)	N/A	N/A	mPSLp, IVIG, CsA	N/A	Withdrawal from HD (durations, N/A)	Alive
Case 2	[44] 2013	56/F	N/A	None	9.05 (serum)	N/A	6 days	mPSLp, PSL, CsA	CMV infection	Withdrawal from HD after 1 month	Alive
Case 3	[45] 2013	47/F	N/A	None	21.9 (serum)	PC-type	17 days	mPSLp, PSL, TCZ	CMV pneumonia	Withdrawal from HD after 2 months	Alive
Case 4	[28] 2016	47/F	None	N/A	4380 (serum) 1600 (CSF)	N/A	1 day	mPSLp, PSL	Respiratory failure requiring mechanical ventilation	Withdrawal from HD after 3 weeks	Alive
Case 5	[28] 2016	76/M	None	ANA	14.1 (N/A)	HV-type	10 days	mPSLp, mPSL sodium succinate	Sepsis (due to systemic, *Staphylococcus aureus* and, CMV infections)	Withdrawal from HD after about 2 weeks	Died
Case 6	[42] 2016	22/M	EBV infection	N/A	N/A	Mixed-type	N/A (about 2 weeks)	TCZ, RTX, ETP	Respiratory failure requiring intubation	Withdrawal from HD (durations, N/A)	Alive
Case 7	[43] 2016	73/M	DM Uveitis	ANA	5.3 (serum)	Biopsy was not performed	N/A (about 2 weeks)	PSL	DVT, DLBCL	Withdrawal from HD after 1 week	Alive
Case 8	[46] 2016	49/F	HTN	ANA	83.4 (serum)	HV-type	4 days	mPSLp, PSL, TCZ	Respiratory failure requiring mechanical ventilation, liver failure, hemorrhage from a rectal ulcer, sepsis due to CNS	Withdrawal from, CHDF and HD, After 2 months	Alive
Case 9	[19] 2016	48/M	None	ANA, anti-SS-A Ab anti-TPO Ab, positive direct Coombs test	16.8 (serum) 945 (pleural effusion)	N/A	42 days	mPSLp, PSL, IVIG, PE, RTX	Cardiogenic shock due to cardiomyopathy	Withdrawal from HD after 2 months	Alive
Case 10	[47] 2016	72/M	HTN, HL	None	46.4 (N/A)	Mixed-type	14 days	PSL, TCZ	Cerebral infarction, necrosis of the ascending colon due to infarction	HDF was continued until the patient’s death	Died
Case 11	[48] 2016	50/M	N/A	Decreased ADAMTS13 activity (9.9%)	2130 (N/A)	HV-type	9 days	mPSLp, PSL, TCZ	N/A	Withdrawal from HD after 3 months	Alive
Case 12	[17], 2017	59/M	DU, Fatty liver	RF	15.2 (N/A)	Biopsy was not performed	N/A (about 3 months)	mPSLp, PSL, TCZ	Bacterial infections (multiple times), PCP, CMV infection, *Candida* bacteremia	Withdrawal from CHDF and HDF after 5 months	Alive
Case 13	[20] 2017	72/M	N/A	Anti-SS-A Ab	26.8 (serum)	Not performed	N/A (about 2 weeks)	mPSLp, PSL, CYA, TCZ	Temporary cardiac arrest, hypovolemic shock and respiratory failure, *Corynebacterium* sepsis and pneumonia	Withdrawal from CHDF and HD after 3 months	Alive
Case 14	[49] 2017	70/M	Esophageal cancer	λ-type BJP, slightly decreased ADAMTS13 activity	33 (serum)	Not performed	21 days	mPSLp, PSL	N/A	Withdrawal from HD after 1 week	Alive
Case 15	[50] 2017	59/M	HBV infection	None	14 (N/A)	Mixed-type	17 days	mPSLp, PSL, TCZ, and CsA	Cardiac arrest possibly caused by massive ascites	Withdrawal from CRRT and HD after 1 month	Alive
Case 16	[51] 2017	38/M	DU	None	26.6 (serum)	HV-type	4 days	mPSLp, PSL, CsA, and TCZ	DIC	Withdrawal from CHDF and HD after 1 month	Alive
Case 17	[52] 2017	24/F	N/A	During pregnancy	842 (serum)	Biopsy was not performed	8 days	mPSLp, PSL	Respiratory failure required mechanical ventilation	Withdrawal from HD after about 3 weeks	Alive
Case 18	[53] 2017	61/F	N/A	anti-CCP Ab	75.9 (serum)	HV-type	7 days	mPSLp, TCZ, RTX	Multiple organ failure requiring mechanical ventilation	Withdrawal from CRRT and HD (durations, N/A)	Alive
Case 19	[33] 2018	50/F	Pityriasis rosea	ANA, anti-SS-A/SS-B Ab, anti-Tg/TPO Ab	15.9 (serum) 5480 (ascites)	Mixed-type	About 20 months after fist admission	mPSLp, PSL, RTX	CMV infection ARDS required mechanical ventilation, Septic shock due to cholecystitis	Withdrawal from CHDF and HD (durations, N/A)	Alive
Case 20	The present case	46/M	None	ANA, anti-SS-A Ab	25.2 (serum) 3310 (ascites)	Mixed-type	50 days	mPSLp, PSL, CsA	Cardiac arrest due to MI, bacterial peritonitis, systemic CMV infections, *Trichosporon asahii* infection in the lungs	CHDF was continued until the patient’s death	Died

*Ab* antibody, *ADAMTS13* A disintegrin and metalloproteinase with a thrombospondin type 1 motif, member 13, *ARDS* acute respiratory distress syndrome, *ANA* antinuclear antibody, *BJP* Bence Jones protein, *CCP* cyclic citrullinated peptide, *CHDF* continuous hemodiafiltration, *CNS* coagulase-negative staphylococci, *CMV* cytomegalovirus, *CRRT* continuous renal replacement therapy, *CsA* cyclosporine A, *CSF* cerebrospinal fluid, *DIC* disseminated intravascular coagulation, *DLBCL* diffuse large B-cell lymphoma, *DU* duodenal ulcer, *DVT* deep vein thrombosis, *EBV* Epstein-Barr virus, *ETP* etoposide, *F* female, *GP* glycoprotein, *HBV* hepatitis B virus, *HD* hemodialysis, *H.pylori Helicobacter* pylori, *HV* hyaline vascular, *IL-6* interleukin 6, *ITP* immune thrombocytopenic purpura, *IVIG* intravenous immunoglobulin, *LN* lymph node, *M* male, *MI* myocardial infarction, *mPSLp* methyl prednisolone pulse therapy, *N/A* not available, *PC* plasma cell, *PCP* pneumocystis pneumonia, *PE* plasma exchange, *RF* rheumatoid factor, *RTX* rituximab, *SS* Sjögren syndrome, *TCZ* tocilizumab, *Tg* thyroglobulin, *TPO* thyroperoxidase, *VEGF* vascular endothelial growth factor

Infection is one of the most serious complications associated with mortality and dilemma during treatment for TAFRO syndrome. Additionally, we should be aware of the coexistence of severe infections during treatment for TAFRO syndrome, including bacterial infection, CMV infection, and fungal infection. To our knowledge, our case is the first case of TAFRO syndrome complicated with *T. asahii* infection. *Trichosporon* species are recognized as a cause of systemic infection in immunocompromised patients [62]. Although *T. asahii* infection was limited to the lungs in the present case, hematologic malignancy is the best-described risk factor for trichosporonosis [62]. As no perforation or injury was observed in the gastrointestinal tract in the autopsy, we suspected that bacterial peritonitis in the present case was caused by bacterial translocation. In this case, a long-term lack of oral intake due to fatigue and venous congestion due to massive ascites were risk factors [63]. In addition, IL-6 hypersecretion might contribute to the increase of intestinal epithelial permeability [64]. As infection itself can lead to the production of proinflammatory cytokines and renal impairment, the early detection of infection is critical to manage TAFRO syndrome.

We could not identify a previous case report of TAFRO syndrome complicated with MI. However, we could identify a few cases that showed an association between hypercytokinemia and cardiomyopathy, which were responsive to immunosuppressive treatment [19, 65, 66]. Interestingly, atherosclerotic lesions in the coronary artery were not observed, and the exact cause of MI was not identified, even after autopsy. We could not ascertain the association between MI and hypercytokinemia. However, embolization associated with hyphae was possibly involved because intravascular invasion of fungal hyphae was observed on histological evaluation of the lung. Interestingly, myocardial calcification was ascertained on microscopic evaluation. We considered that the myocardial calcifications corresponded to scattered high intensity lesions in CT images, which revealed remarkable progression within a short period (Fig. 4b–d). These lesions might contribute to the impairment of cardiac muscle contraction. Myocardial calcifications could be caused by a variety of etiologies [67], but they are largely divided into dystrophic metastatic myocardial calcification, which were described by Gore and Arons [68]. Dystrophic myocardial calcification is usually caused by local tissue damage and cellular necrosis irrespective of serum calcium levels and calcium homeostasis [69]. Metastatic myocardial calcification represents the sequelae of hypercalcemia and/or abnormal calcium homeostasis, and it is most commonly observed in patients with end-stage renal disease on HD [69]. In patients with sepsis, sepsis-induced cytokines and cardiosuppressing circulating mediators, alterations of calcium flux in myocytes (intracellular calcium overload), and mitochondrial dysfunction are thought to be related to the pathophysiology of cardiac dysfunction (septic cardiomyopathy) [70]. Septic cardiomyopathy can result in diffuse myocardial calcification due to the pathophysiology of dystrophic myocardial calcification [71]. However, diffuse myocardial calcification in patients with severe sepsis is rare [72], and it is sometimes accidentally detected on chest CT [73]. The term sepsis-related myocardial calcification was used by van Kruijsdijk et al. [74]. Calcification mainly involves the left ventricle, but the right ventricle can be involved as well [73], which is possibly associated with the risk of sudden cardiac death [75]. Although renal impairment was apparent in the present case, we considered that calcifications in the myocardial cells and skeletal muscle cells of the diaphragm without apparent vascular calcification could not be explained by only short-term uremic conditions. Additionally, hypercalcemia was never observed during treatment in the present case. Because myocardial calcification was observed in non-damaged myocardial cells, we suspected sepsis-related myocardial calcification in the present case. As sepsis-induced excessive cytokine production is one cause of septic cardiomyopathy [70], we considered that hypercytokinemia due to TAFRO syndrome might be partially associated with the exacerbation of cardiac dysfunction in the present case.

## Conclusions

We encountered a case of severe TAFRO syndrome unresponsive to commonly used combination therapies involving glucocorticoids and CsA. Multiple severe infections and MI contributed to death in the present case. In this case, myocardial calcification became evident within a short period, and sepsis-related myocardial calcification was suspected. We should consider the risk of severe infections and cardiac dysfunction in patients with TAFRO syndrome during the clinical course. Further studies are required to establish a more appropriate treatment strategy for TAFRO syndrome and indicators of clinical response. Interestingly, autopsy results in the present case showed that MCD-like histopathological findings were responsive to immunosuppressive therapy; however, renal impairment, anasarca, and ascites were not responsive. Based on the autopsy results, fibrotic changes in mediastinal fat tissue could be histopathological manifestations of TAFRO syndrome.

## Abbreviations

ALP: Alkali phosphatase
CAG: Coronary angiography
CD: Castleman disease
CMV: Cytomegalovirus
CRP: C-reactive protein
CRRT: Continuous renal replacement therapy
CsA: Cyclosporine A
CT: Computed tomography
FDG-PET: ^18^F–fluorodeoxyglucose positron emission tomography
FE_UN_: Fractional excretion of urea nitrogen
GFR: Glomerular filtration rate
HD: Hemodialysis
HHV-8: Human herpesvirus 8
HV: Hyaline vascular
IgG: Immunoglobulin G
IL: Interleukin
iMCD: Idiopathic multicentric Castleman disease
IP-10: Interferon γ-induced protein 10 kDa
IPL: Idiopathic plasmacytic lymphadenopathy
mPSL: Methyl prednisolone
NOS: Not otherwise specific
PC: Plasma cell
POEMS: Polyneuropathy, organomegaly, endocrinopathy, monoclonal gammopathy, and skin changes
ROSC: Return of spontaneous circulation
SjS: Sjögren syndrome
SLE: Systemic lupus erythematosus
STEMI: ST-elevated myocardial infarction
TAFRO: Thrombocytopenia, anasarca, fever, reticulin myelofibrosis/renal failure, and organomegaly
TCZ: Tocilizumab
TMA: Thrombotic microangiopathy
UCD: Unicentric Castleman disease
VEGF: Vascular endothelial growth factor
WBC: White blood cell

## Authors' information

There are two Keisuke Kamiyas associated with this manuscript. They are spelled the same in English but different in Japanese.
